# Vaping-Related Clotting Phenomena Presenting As Central Retinal Vein Occlusion

**DOI:** 10.7759/cureus.27700

**Published:** 2022-08-05

**Authors:** Alexander M Balinski, Rachel N Harvey, Ryan B Ko, Melanie M Smalley, Nathan E Cutler, Maham T Siddiqi

**Affiliations:** 1 Internal Medicine, Oakland University William Beaumont School of Medicine, Rochester, USA; 2 Internal Medicine, Beaumont Health, Royal Oak, USA; 3 Ophthalmology, Retina Consultants of Michigan, Southfield, USA

**Keywords:** covid-19, hypercoagulation, roth spots, hematologic and clotting, central retinal vein occlusion (crvo), effects of vaping

## Abstract

Central retinal vein occlusion (CRVO) typically manifests as unilateral vision loss from thrombosis and occlusion of the central retinal vein in patients with thrombophilic risk factors. Here we report a case of a 23-year-old male with three weeks of intermittent left-sided eye pressure and vision loss, who was found to have decreased visual acuity, retinal hemorrhages, and an impending CRVO in his left eye. Upon further evaluation, infectious disease and autoimmune labs were normal, but he had mildly increased right heart pressures and hypercoagulable changes in the right middle cerebral artery. He denied any personal or family history of clotting disorders but noted a four-year history of vaping. He was started on anticoagulation and discharged. Outpatient genetic testing for Factor V Leiden, protein C, protein S, and prothrombin G20210 was normal. His visual acuity returned to normal in the left eye and the retinal hemorrhages resolved. After the exclusion of organic causes, significant vaping history was considered the likely etiology of his hypercoagulable state and resultant CRVO. Vaping-related clotting phenomena may explain the etiology of an otherwise unexplained CRVO, but further investigation of the long-term health consequences of electronic cigarette use is still needed.

## Introduction

Central retinal vein occlusion (CRVO) typically manifests as unilateral vision loss from thrombosis and occlusion of the central retinal vein in older patients with hypercoagulable and thrombophilic risk factors such as hypertension, hypercholesterolemia, and diabetes mellitus. Other known etiologies include primary inflammatory or degenerative venous wall disease and/or central retinal vein compression by an adjacent sclerotic artery [1]. Our current understanding of CRVO in younger patients is limited, but studies suggest that this population may present with nontraditional risk factors and experience different outcomes [2]. Here we report an unusual case of CRVO likely secondary to vaping-related clotting phenomena in a young adult male. This article was previously presented as a meeting abstract at the 2021 American College of Physicians (ACP) Michigan Chapter Virtual Residents/Medical Students Day on May 7, 2021.

## Case presentation

**Figure 1:**
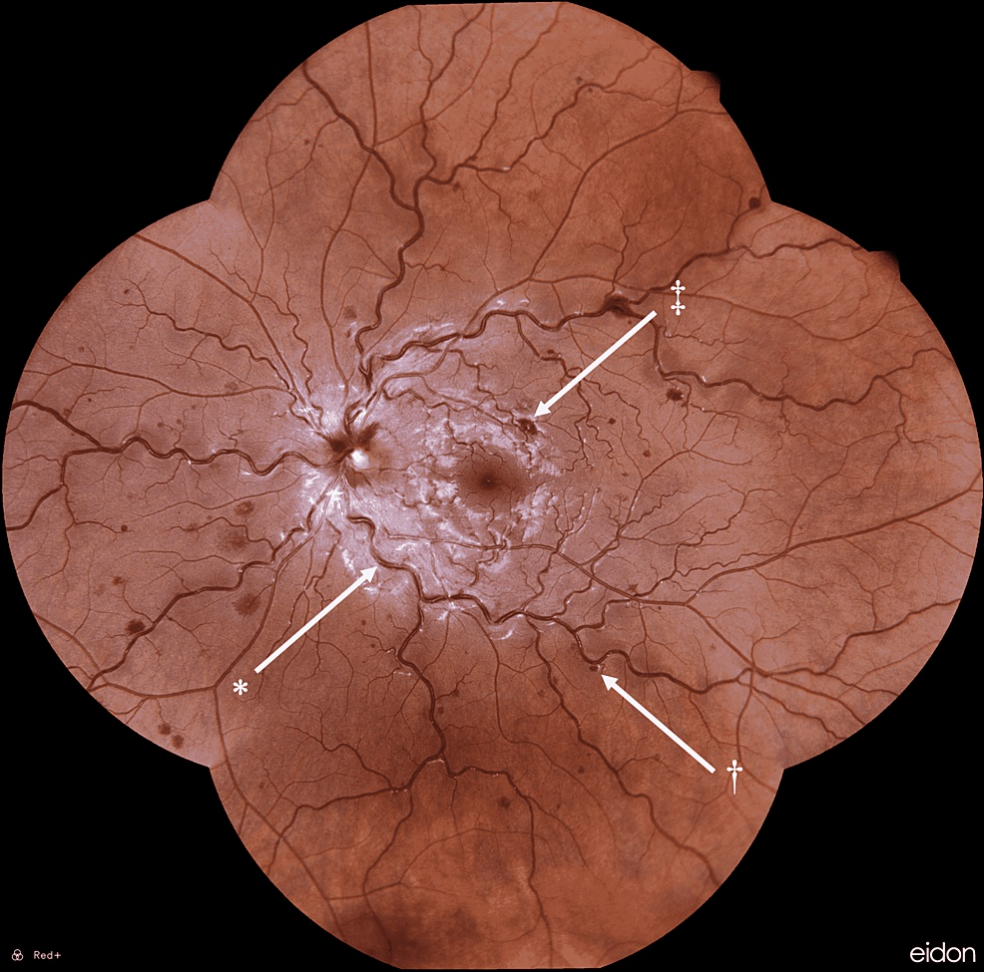
Fundoscopy demonstrating *dilated and tortuous venous vessels, †moderate dot/blot hemorrhages, and ‡Roth spots

A 23-year-old male presented to the emergency department with three weeks of intermittent left-sided eye pressure and vision loss. He had been sent by his retina specialist for further evaluation of multiple Roth spots and an impending CRVO in his left eye with an associated decrease in visual acuity (Figure 1). Past medical history included sports-induced asthma and self-reported Raynaud’s phenomenon. He denied any history of coronavirus disease 2019 (COVID-19) vaccination. He also denied any personal or family history of clotting disorders and tobacco use but noted a four-year history of electronic cigarette usage, which had increased over the past several months due to stress and anxiety surrounding the COVID-19 pandemic. He was unable to provide any information regarding specific vaping product use at that time.

**Figure 2:**
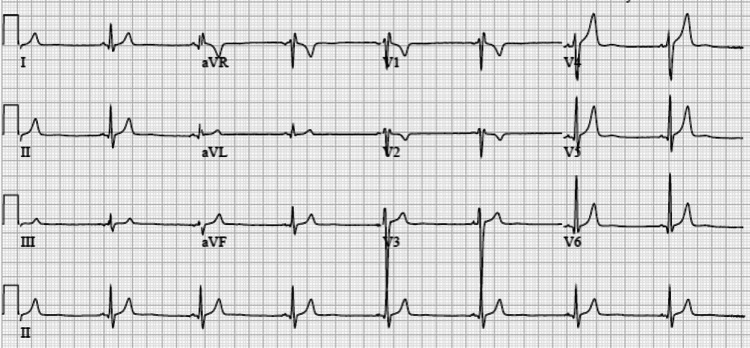
Electrocardiogram demonstrating sinus bradycardia and incomplete right bundle branch block

**Figure 3:**
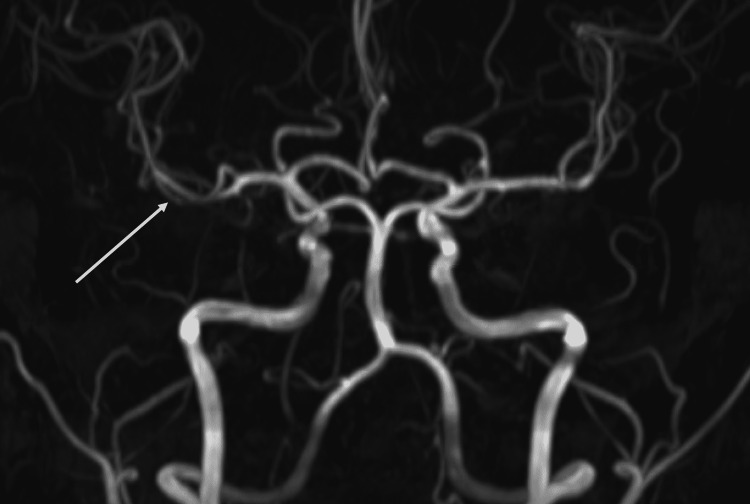
Brain magnetic resonance angiography showing possible hypercoagulable changes in the M2 branch of the right middle cerebral artery (shown)

On exam, vital signs were normal, but he was notably anxious with a mildly delayed capillary refill in his bilateral feet and was confirmed to have an impending CRVO on a left-sided ocular exam. His right-sided ocular exam was normal. Visual acuity was 20/25 in the left eye and 20/20 in the right eye. His labs showed a leukocyte count of 11.9 bil/L but were otherwise unremarkable. COVID-19, cryoglobulin, immunoglobulin, hepatitis panel, human immunodeficiency virus (HIV), herpes simplex virus (HSV), rapid plasma reagin (RPR), erythrocyte sedimentation rate (ESR), C-reactive protein (CRP), as well as autoimmune labs including antinuclear antibody (ANA), antineutrophil cytoplasmic antibody (ANCA), antiphospholipid antibody, centromere antibody, histidyl-tRNA synthetase (Jo-1), paroxysmal nocturnal hemoglobinuria (PNH), rheumatoid factor, and topoisomerase I (Scl 70) were obtained and resulted normal. An electrocardiogram showed incomplete right bundle branch block (Figure 2), and a transthoracic echocardiogram was negative for vegetation but demonstrated mild right ventricular enlargement with mildly increased right heart pressures, concerning for pulmonary hypertension. Brain magnetic resonance angiography was obtained and showed possible hypercoagulable changes in the M2 branch of the right middle cerebral artery (Figure 3).

He was started on anticoagulation and discharged for outpatient follow-up. Genetic testing for Factor V Leiden, protein C, protein S, and prothrombin G20210 was conducted in the outpatient setting but returned as normal. Subsequent follow-up demonstrated no symptomatic recurrence, and prophylactic anticoagulation was maintained. His visual acuity returned to 20/20 in the left eye and the retinal hemorrhages resolved.

## Discussion

Central retinal vein occlusion commonly presents with new-onset blurred or distorted vision due to impaired retinal venous return and macular edema. Common patient risk factors for CRVO include advanced age, diabetes, and hypertension but genetic etiologies such as Factor V Leiden mutation, hyperprothrombinemia, and protein C or S deficiency should be suspected for presentations in younger individuals [3]. Social factors such as smoking tobacco and electronic cigarette use should also be considered as possible etiologies of an underlying hypercoagulable state in these non-traditional demographics. 

Smoking tobacco has historically been a significant risk factor for hypercoagulability, enacting its toxic effects through induction of endothelial damage and promotion of the coagulation cascade [4]. Currently, electronic cigarettes have replaced smoking tobacco as the most popular vehicle for nicotine use by adolescents [5]. Although there is limited data regarding the long-term health effects of vaping, chronic electronic cigarette use has been shown to increase the risk of thrombogenic events through increased platelet aggregation, upregulated adhesion receptors, and impaired vascular elasticity [6,7]. Additionally, the use of electronic cigarette aerosol with nicotine has been shown to significantly increase heart rate, increase arterial stiffness, and obstruct conducting airways [8]. Chronic increases in heart rate may exacerbate structural and functional changes in arterial walls, resulting in an increased risk of cardiovascular disease [9].

In this case, our patient was a young adult male with no significant past medical or family history, which prompted initial diagnostic evaluation for possible autoimmune and hypercoagulable factors as etiologies of his clinical presentation. His lab findings were non-specific, but diagnostic imaging demonstrated vascular brain and right heart changes suggestive of underlying hypercoagulability. After thorough exclusion of organic causes, his significant vaping history was considered the likely etiology of this hypercoagulable state and resultant CRVO. 

A significant limitation of this case is the inability to definitively rule out current or prior COVID-19 infection associated with the patient’s clinical presentation. Recently documented cases of CRVO in young adults have been associated with COVID-19 [10,11]. Though typically COVID-19 positive on initial testing, these cases can be associated with false-negative tests revealed as positive upon follow-up COVID-19 antibody testing [11]. In this case, clinical suspicion of COVID-19 was low as the patient denied any COVID-like symptoms or recent sick contacts, and initial COVID-19 testing was negative upon hospital admission. However, repeat testing was not conducted, and antibody testing was not attempted upon subsequent outpatient follow-up. Therefore, COVID-19, while less likely, still cannot be definitively ruled out as the underlying etiology of CRVO in this case.

## Conclusions

This case highlights the importance of a thorough history and diagnostic workup when considering etiologies of hypercoagulability in otherwise healthy patients presenting with CRVO. Significant vaping history may explain the etiology of otherwise unexplained CRVO, but there is a need for further investigation of the long-term health consequences of electronic cigarette use in the adolescent and adult population.
